# Cholera in Buffalo

**Published:** 1850-10

**Authors:** 


*Cholera in Buffalo.*—Our city has, Providentially, escaped a visitation of Epidemic Cholera during the past summer. Single cases have occurred from time to time, but the disease has not prevailed to an extent sufficient to entitle it to be called an epidemic. We hope to give hereafter some account of the few cases that have been observed.

Dysentery has been the disease most commonly met with in practice during the summer, as it is at the present time. In general this disease has not assumed a severe form, but in some instances it has been characterized by hemorrhagic discharges which have proved rapidly fatal. Dysentery has prevailed, as we are informed, more in the vicinity of Buffalo than in the city.

